# Mo_2_C-induced hydrogen production enhances microbial electrosynthesis of acetate from CO_2_ reduction

**DOI:** 10.1186/s13068-019-1413-z

**Published:** 2019-04-01

**Authors:** Shihao Tian, Haoqi Wang, Zhiwei Dong, Yang Yang, Hao Yuan, Qiong Huang, Tian-shun Song, Jingjing Xie

**Affiliations:** 1 State Key Laboratory of Materials-Oriented Chemical Engineering, Nanjing Tech University, Nanjing, 211816 People’s Republic of China; 2 College of Life Science and Pharmaceutical Engineering, Nanjing Tech University, Nanjing, 211816 People’s Republic of China; 3 Jiangsu Branch of China Academy of Science & Technology Development, Nanjing, 210008 People’s Republic of China; 4 Jiangsu Collaborative Innovation Center of Atmospheric Environment and Equipment Technology, Jiangsu Key Laboratory of Atmospheric Environment Monitoring and Pollution Control (AEMPC), Nanjing University of Information Science & Technology, Nanjing, 210044 People’s Republic of China; 5 Jiangsu National Synergetic Innovation Center for Advanced Materials (SICAM), Nanjing, 211816 People’s Republic of China

**Keywords:** Microbial electrosynthesis, Carbon dioxide, Indirect electron transfer, Hydrogen evolution reaction, Molybdenum carbide

## Abstract

**Background:**

Microbial electrosynthesis (MES) is a biocathode-driven process, in which electroautotrophic microorganisms can directly uptake electrons or indirectly via H_2_ from the cathode as energy sources and CO_2_ as only carbon source to produce chemicals.

**Results:**

This study demonstrates that a hydrogen evolution reaction (HER) catalyst can enhance MES performance. An active HER electrocatalyst molybdenum carbide (Mo_2_C)-modified electrode was constructed for MES. The volumetric acetate production rate of MES with 12 mg cm^−2^ Mo_2_C was 0.19 ± 0.02 g L^−1^ day^−1^, which was 2.1 times higher than that of the control. The final acetate concentration reached 5.72 ± 0.6 g L^−1^ within 30 days, and coulombic efficiencies of 64 ± 0.7% were yielded. Furthermore, electrochemical study, scanning electron microscopy, and microbial community analyses suggested that Mo_2_C can accelerate the release of hydrogen, promote the formation of biofilms and regulate the mixed microbial flora.

**Conclusion:**

Coupling a HER catalyst to a cathode of MES system is a promising strategy for improving MES efficiency. 
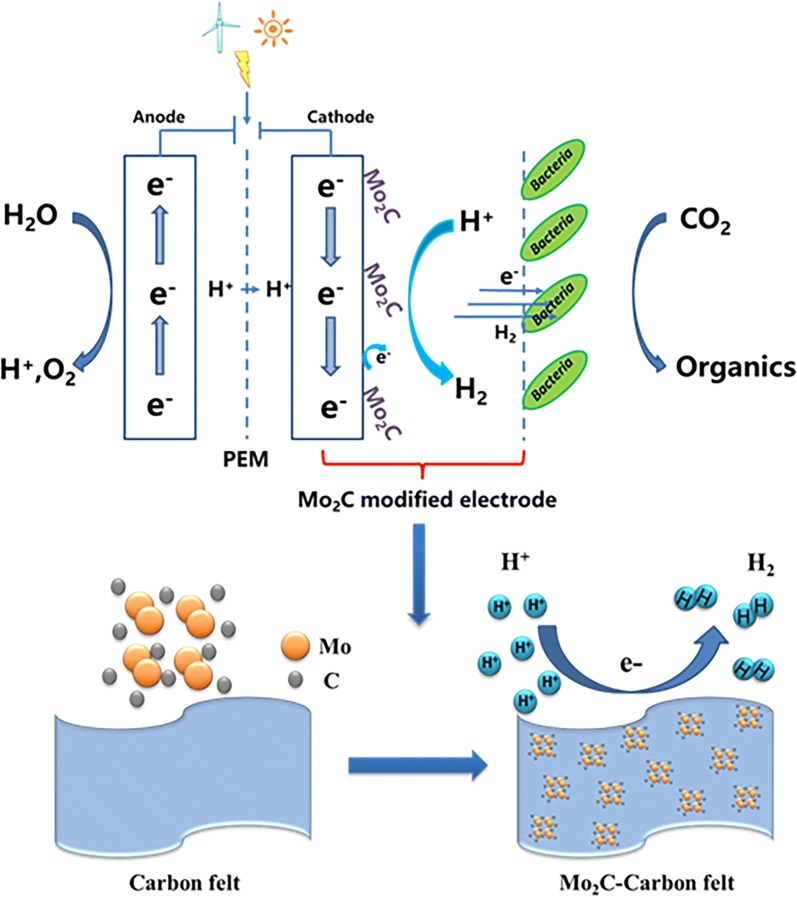

**Electronic supplementary material:**

The online version of this article (10.1186/s13068-019-1413-z) contains supplementary material, which is available to authorized users.

## Background

Due to the fossil resources depletion, the population expansion and continuously economy prosperity, there are increasing demands for more clean, affordable, reliable and sustainable energy supplies [1]. With the more competitive cost, additional engagement of the scientific, financial and public–policy communities, the renewable energy supply from hydropower, solar cells and wind turbines might power the global economic growth, increase energy security, as well as mitigate the risks of climate change [1]. However, due to the energy intermittency of renewable sources, efficient energy storage is a prerequisite for a smooth transition to a clean energy economy [2]. Among existing renewable energy storage technologies, microbial electrosynthesis (MES) is an emerging strategy [3]. MES is a biocathode-driven process, in which the electroautotrophic microorganisms may use electrons generated from renewable energy on the cathode as energy sources and CO_2_ as only carbon source to produce chemicals [4, 5]. Thus, the hard-to-store energy generated from renewable sources is converted into high value-added chemicals [6, 7].

The operation efficiency of MES is highly affected by the activity of the biocatalysts and the supply of electrons from the cathode. Therefore, a large number of studies on MES have been focused on the screening and optimization of electroautotrophic microorganisms [8] or the improvement of the electron transfer capability from electrodes to biocatalysts by materials science [7, 9]. Biocatalysts used in MES systems can be classified into two major categories: pure bacterium [3, 10–13] and mixed microbial flora [8, 14–20]. Although biocatalysts used in MES were different, the dominant product in MES was acetate for early years of MES development. Recently, two independent research groups screened and obtained a large number of mixed bacterial populations that are capable of synthesizing isopropanol [19] and butyric acid [20], thereby enabling MES systems to synthesize C3 and C4 compounds. A lot more MES studies focused on the modification of electrode materials to improve the electron transfer rate and biocompatibility of the electrodes in the MES system [21, 22]. Decoration of conductive materials on both carbon cloth [10, 11] and three-dimensional scaffold [12–14, 16, 23], such as RVC or nickel foam, may increase the acetate production rate as well as CO_2_ to acetate conversion rates.

However, most of the existing electrode modification has been focused on direct electron transfer (DET). In DET, electroautotrophic microbes may obtain electrons that pass through membrane proteins, such as C-type cytochromes and H^+^-dependent Rnf complex or hydrogenase [24]; or electrons are directly obtained from the cathode via biological nanowires [25], i.e., conductive *pili* [9, 21, 26]. Different from the DET, which requires direct contact between microbes and the cathode, the mediated electron transfer (MET) is accomplished through the redox electron mediator secreted by the autotrophic microorganisms or extracellular H_2_, formate, Fe^2+^, NH_3_, etc. [9]. However, the researches on MES system through MET were much less compared to DET. Liao’s group and Nocera’s group demonstrated examples of electrochemically converting electrons into electron mediators, such as formic acid and hydrogen, respectively [27, 28]. Therefore, the engineered *Ralstonia eutropha*, which cannot obtain electrons directly from electrodes, may biotransfer CO_2_ and electron mediators into biofuels [27, 28].

Most electroautotrophic microorganisms used in MES (either pure bacteria or the dominant strains in mixed microbial flora) are acetogens. Acetogens adopt the Wood–Ljungdahl pathway to fix CO_2_ with the reducing power provided by H_2_. In MES, H_2_ might be produced in the cathodic chamber by water electrolysis; however, the activation energy of hydrogen evolution reaction without catalyst is relatively high. Therefore, decorating H_2_ evolution catalysis on the cathode of MES and using hydrogen as an electron mediator to improve electron transfer from the electrode to the electroautotrophic microorganisms should be an effective strategy for improving MES efficiency [21].

The common method of producing hydrogen is to electrochemically split water into hydrogen and oxygen gases [29]. Therefore, substantial research was applied to develop catalysis materials for hydrogen evolution reaction (HER), reduce kinetic barriers, and perform efficient electrolysis [30]. The most active HER electrocatalysts are obtained from precious platinum group metals, but they are too expensive for large-scale deployment. Among the most frequently proposed HER electrocatalysts, transition metal carbides are considered as promising replacements for platinum cathode catalyst for HER due to their high catalytic activities, low cost, and high abundance [31]. In this study, we coupled an active HER electrocatalyst, i.e., molybdenum carbide (Mo_2_C), to a microbial electrosynthesis system to demonstrate a highly effective CO_2_ reduction process through chemical and biological catalyses.

## Results

### Characterization of electrocatalysts

X-ray diffraction (XRD) analysis was used to confirm the crystallinity and phase structure of the commercially available Mo_2_C powder. The XRD patterns (Additional file 1: Fig S1) showed that all the diffraction peaks of the Mo_2_C sample are unambiguously indexed based on the hexagonal of β-Mo_2_C phases (PDF#35-0787). No evident diffraction peaks of impurities were detected, and strong diffraction peaks were monitored at 2–θ angles of 39.3°, 69.5°, and 4.6°, which can be assigned to the (101), (103), and (112) diffraction planes, respectively, of Mo_2_C.

**Fig. 1:**
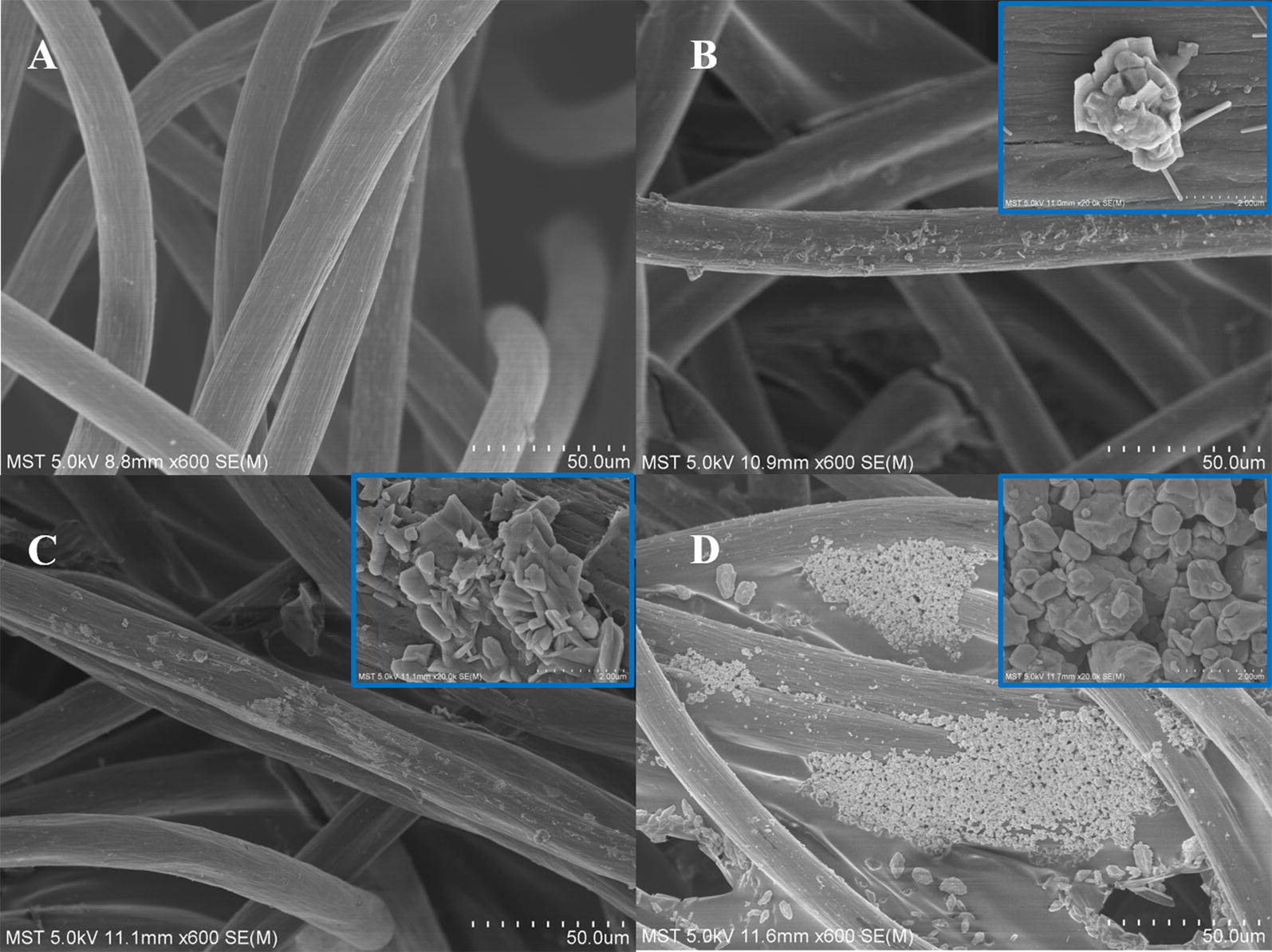
SEM images of **a** CF, CF with **b** 2 mg cm^−2^ Mo_2_C, **c** 4 mg cm^−2^ Mo_2_C and **d** 12 mg cm^−2^ Mo_2_C

As observed from the scanning electron microscope (SEM) image (Fig. 1), the morphology and structure of Mo_2_C–CF and CF were investigated. The surface of bare CF was smooth, whereas the CF samples with different amounts of Mo_2_C loading presented distinct morphology. CF with 12 mg cm^−2^ Mo_2_C (Fig. 1d) exhibited good particle distribution and loading conditions. Moreover, the elemental analysis of the selected region (Additional file 1: Table S1) showed the distribution of the elements on the cathode materials compared with that of the control group, which demonstrated that Mo_2_C was successfully adsorbed onto CF.

### Electrocatalytic measurements

**Fig. 2:**
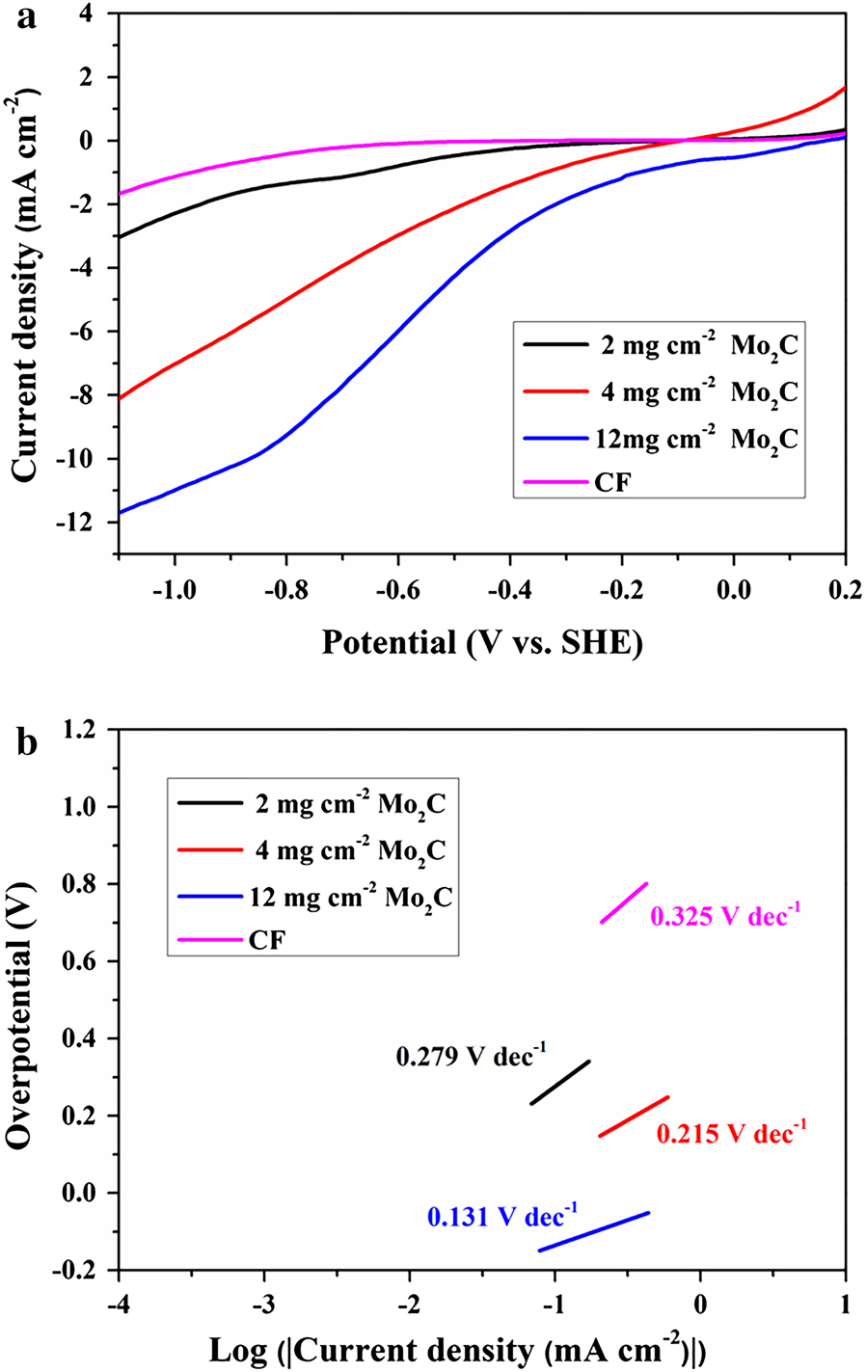
LSV (**a**) and corresponding Tafel plots (**b**) of Mo_2_C–CF and CF in cathode medium

The electrocatalytic performance of Mo_2_C–CF for HER was then evaluated in a cathode medium using a three-electrode system. Bare CF was used as the control group. During the test, Fig. 2a shows that CF delivered poor performance for linear sweep voltammetry (LSV) curves shifting from 0.2 to − 1.2 V (vs. SHE). As expected, the HER performance of CF can be considerably enhanced by 12 mg cm^−2^ Mo_2_C modification with a large overpotential of only − 0.173 V at a representative current density of 1 mA cm^−2^ (η1). The overpotential of CF with 4 mg cm^−2^ and 2 mg cm^−2^ Mo_2_C modification was − 0.333 V and − 0.663 V at 1 mA cm^−2^ (η1), respectively. Tafel plots were also obtained from the LSV curves. As shown in Fig. 2b, the Tafel plots constructed from steady-state polarization measurements presented HER activity from a kinetic viewpoint. For comparison, the Tafel slope of 12 mg cm^−2^ Mo_2_C was 0.131 V dec^−1^, which was lower than those of 4 mg cm^−2^ Mo_2_C (0.215 V dec^−1^), 2 mg cm^−2^ Mo_2_C (0.279 V dec^−1^), and the control (0.325 V dec^−1^). Among the samples, CF with 12 mg cm^−2^ achieved the highest electrocatalytic performance for generating hydrogen from a cathode medium.

**Fig. 3:**
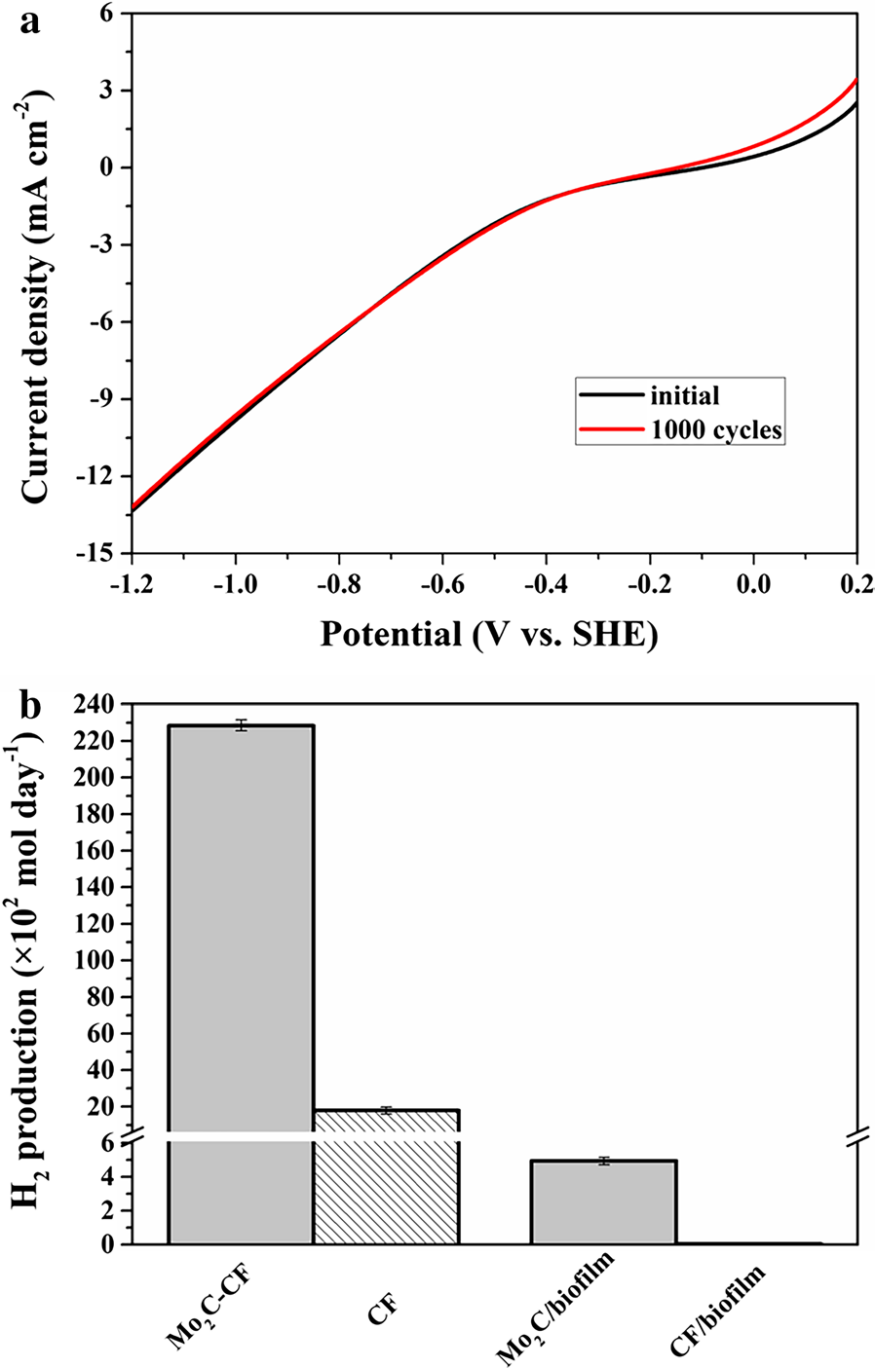
LSV of Mo_2_C–CF before and after 1000 CV cycles in cathode medium (**a**) and average H_2_ production rate for bare Mo_2_C–CF, CF, Mo_2_C biofilm (on day 14) and CF biofilm (on day 14) in cathode medium (**b**)

The hydrogen production by Mo_2_C–CF and CF in the MES system was experimentally quantified by gas chromatography (GC), which confirmed the same trend. The average H_2_ production rate of Mo_2_C–CF (12 mg cm^−2^) reached about 2.29 × 10^4^ mol day^−1^, which was 12.7 times than that of CF (1.80 × 10^3^ mol day^−1^) (Fig. 3b). It showed that the Mo_2_C modification significantly improved the hydrogen evolution in the MES system.

Furthermore, the stability of Mo_2_C–CF (12 mg cm^−2^) was evaluated after 1000 cycles of cyclic voltammograms (CV) scans in the cathodic medium. There was no significant difference between LSV curves before and after CV scans (Fig. 3a). The results indicated that the Mo_2_C-decorated CF was quite stable in this system.

### MES of acetate from CO_2_

**Fig. 4:**
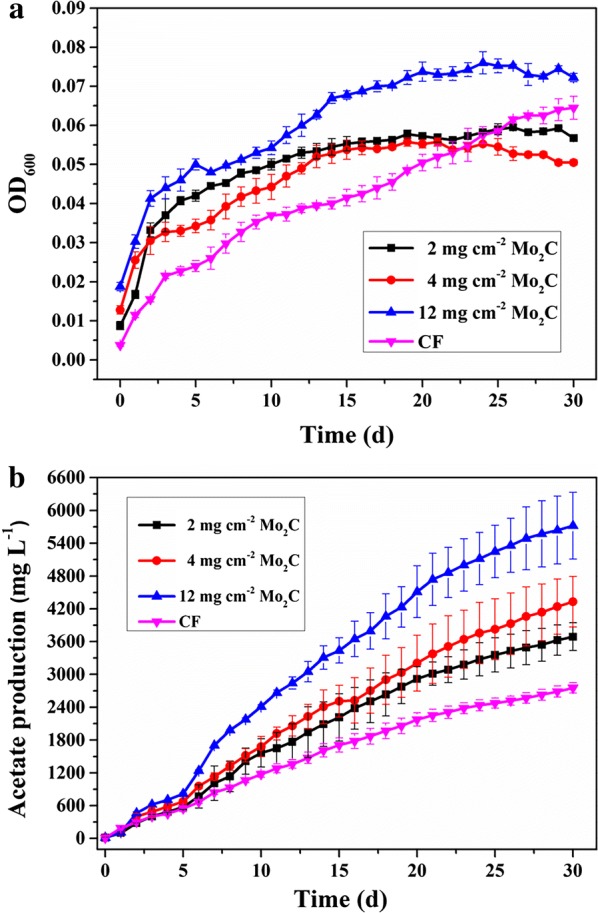
OD_600_ (**a**) and acetate production (**b**) of MES with Mo_2_C–CF and CF over 30 days at − 0.85 V vs. SHE

After inoculation, the OD_600_ of the cathodic medium represents microorganism growth in the solution to a certain extent. The OD_600_ values of the MES with Mo_2_C and the control were approximately 0.02 after inoculation (Fig. 4a). The OD_600_ value of the MES with 12 mg cm^−2^ Mo_2_C increased during the first 12 days of operation, and then increased slowly, and finally stabilized. The maximum OD_600_ value reached 0.07 ± 0.01, which was still higher than that of the control. However, the final OD_600_ values of the MES with 4 mg cm^−2^ and 2 mg cm^−2^ Mo_2_C were slightly lower than that of the control, reaching 0.05 ± 0.01 and 0.06 ± 0.01, respectively. In general, the growth rate of the OD_600_ of MES with Mo_2_C was higher than that of the control at the beginning of the experiment. However, the differences in the OD_600_ values became insignificant after the OD_600_ value had stabilized.

Acetate was the predominant product in all the MES reactors (Fig. 4b). Tiny amount of other products such as formate and ethanol was observed in the initial 7 days and gradually declined (Additional file 1: Fig S2). After 30 days, an increase in acetate production was observed in all the MES groups. However, the acetate production rate increased with an increase in Mo_2_C loading. After a 30-day operation, the MES with 12 mg cm^−2^ Mo_2_C obtained the highest maximum acetate titer of 5.72 ± 0.6 g L^−1^, followed by the MES with 4 mg cm^−2^ and 2 mg cm^−2^ Mo_2_C, which yielded a maximum acetate titer of 4.33 ± 0.5 g L^−1^ and 3.69 ± 0.3 g L^−1^, respectively. The maximum acetate titer of the control was only 2.75 ± 0.10 g L^−1^. Meanwhile, the average acetate production rate of the MES with 12 mg cm^−2^ Mo_2_C was the highest (0.19 ± 0.02 g L^−1^ day^−1^), i.e., 2.1 times higher than that of the control (0.09 ± 0.01 g L^−1^ day^−1^). The acetate production rates of the MES with 4 mg cm^−2^ and 2 mg cm^−2^ Mo_2_C were 0.14 ± 0.02 g L^−1^ day^−1^ and 0.12 ± 0.01 g L^−1^ day^−1^, respectively.

**Fig. 5:**
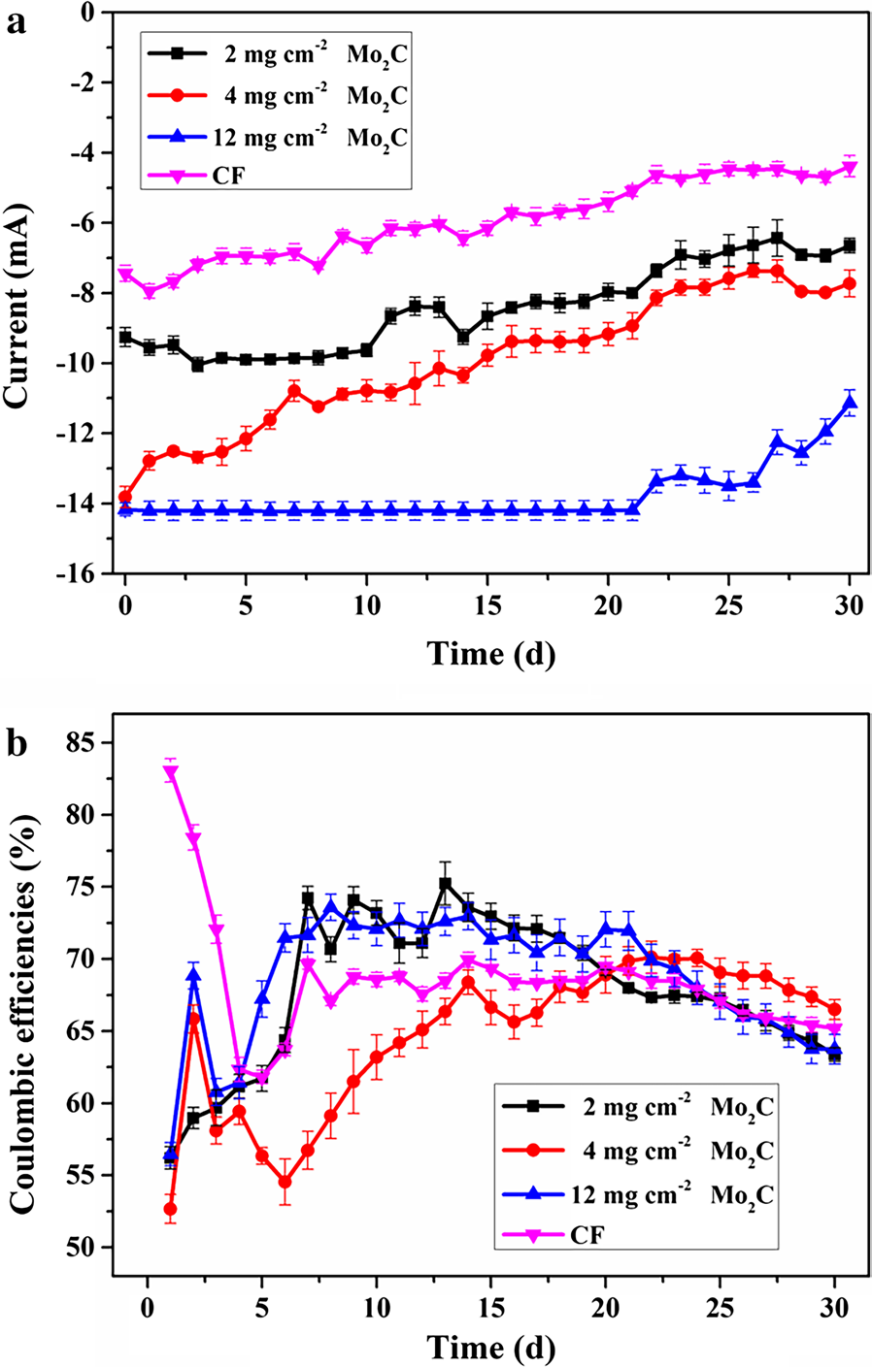
Current (**a**) and CEs (**b**) of MES with Mo_2_C–CF and CF over 30 days at − 0.85 V vs. SHE

Current was generated immediately after inoculation (Fig. 5a). The current of the MES with 12 mg cm^−2^ Mo_2_C was stable for the first 22 days; however, those of the other MES systems started to decline after 7 days. By the end of the experiment, the current of the control was only 5.82 ± 0.2 mA, which was lower than those of the MES with 2, 4, and 12 mg cm^−2^ Mo_2_C (8.75 ± 0.3, 9.90 ± 0.2, and 13.1 ± 0.3 mA, respectively). The overall trend of the current in all the MES systems presented a slow decline. This phenomenon is similar to those in other reports [10, 11, 32, 33]. Furthermore, coulombic efficiency (CE) was calculated (Fig. 5b). The CEs of the control and MES with Mo_2_C were close and maintained a value of approximately 64 ± 0.7%.

### Bioelectrocatalytic activity

**Fig. 6:**
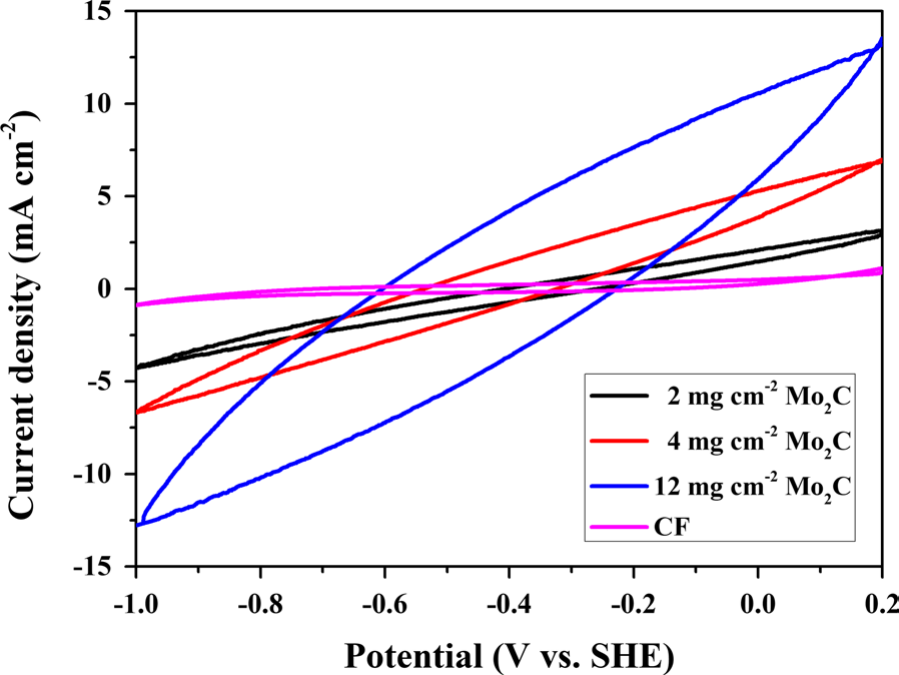
CV of MES with Mo_2_C–CF and CF on day 14 of the MES operation vs. SHE

To investigate the electrochemical behavior of the biocathodes, CV was performed in the MES reactors on day 14 of MES operation (Fig. 6). The visualized maximum current of the control biocathode in forward (1.11 mA cm^−2^) and reverse (− 0.88 mA cm^−2^) scans was lower than that of the Mo_2_C biocathode. Apparently, the MES with 12 mg cm^−2^ Mo_2_C (13.53 mA cm^−2^ and − 12.78 mA cm^−2^) exhibited a higher cathodic current density than the other systems, whereas the control yielded the lowest cathodic current density. The results indicated that the electron transfer capability of the cathode was improved by Mo_2_C modification. In addition, the MES with 12 mg cm^−2^ Mo_2_C obtained the highest electron transfer ability.

On day 14 of MES operation, the average H_2_ production rates in MES with Mo_2_C-biofilm (12 mg cm^−2^) and CF biofilm were measured, respectively. The average H_2_ production rate of Mo_2_C biofilm reached about 4.94 × 10^2^ mol day^−1^, while the H_2_ production was almost undetectable in the CF biofilm (Fig. 3b). The H_2_ production in MES with Mo_2_C-modified biocathode was significantly higher than that in MES without modification. The observation of lower hydrogen production rate in biocathode compared with the bare cathode might be due to the constant consumption of hydrogen by microorganisms in the MES system.

### Biofilm analyses

The surfaces of the biofilms were analyzed at the end of the experiment via SEM (Additional file 1: Fig. S3). The surface morphology of the cathode from the control group was relatively smooth, and thin biofilm could be observed on the carbon fiber. CF with Mo_2_C modification contained thick biofilm on the carbon fiber. An increasing number of microorganisms attached to the cathode with an increase in catalyst loading. The protein content of the cathode was measured at the end of experiments. The protein contents of the MES with 2, 4, and 12 mg cm^−2^ Mo_2_C were 220.53 ± 2.54 μg cm^−2^, 226.81 ± 2.11 μg cm^−2^, and 336.39 ± 0.42 μg cm^−2^, respectively. The MES with 12 mg cm^−2^ Mo_2_C obtained the highest protein content, which was approximately 2.1 times higher than that of the control (163.76 ± 6.08 μg cm^−2^). The protein content results are consistent with the SEM observation and indicated that the difference in biomass may be caused by acetate production change.

**Fig. 7:**
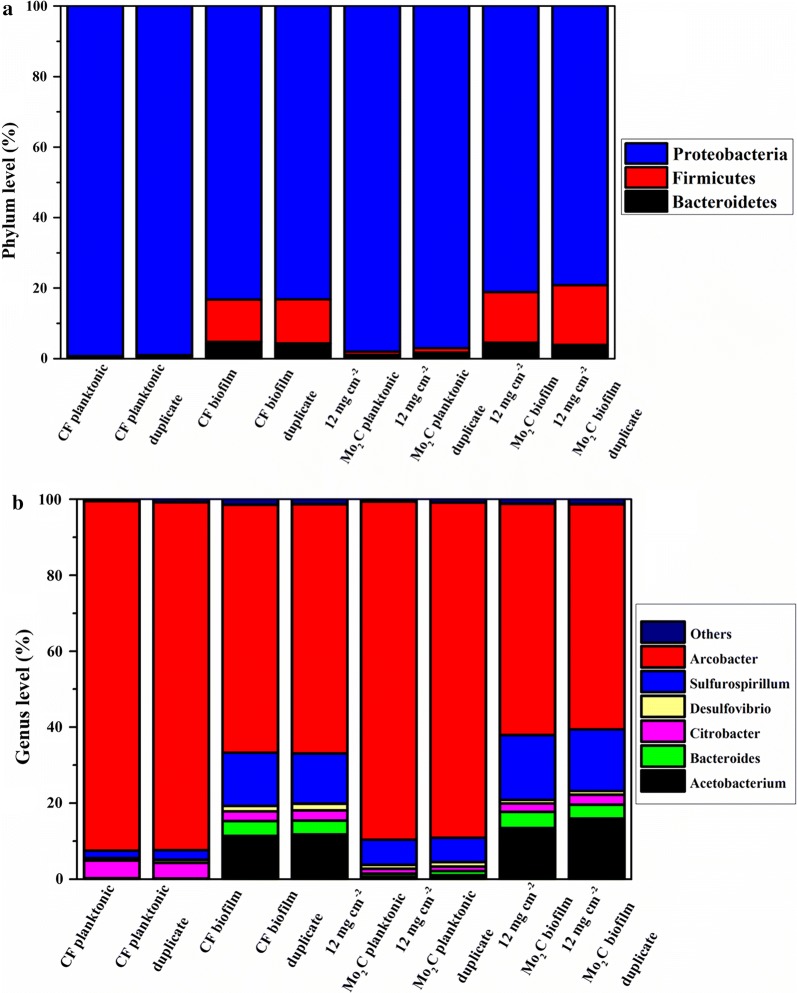
At the end of the experiments, the relative abundance of bacterial phylum level (**a**) and genus level (**b**) in biofilms and planktonic cells from two replicate reactors with 12 mg cm^−2^ Mo_2_C–CF and CF. Others represent the sum of bacteria in a sample with a relative abundance of < 1%

Furthermore, the microbial community of planktonic cells and biofilms in the control and MES with 12 mg cm^−2^ Mo_2_C groups were analyzed at the end of the experiments. At the phylum level (Fig. 7a), the relative abundance of *Proteobacteria*, *Bacteroidetes*, and *Firmicutes* was dominant. *Proteobacteria* was the most abundant phylum in planktonic cells and biofilms in the control and MES with 12 mg cm^−2^ Mo_2_C groups. Simultaneously, *Proteobacteria* accounted for over 98 ± 1% of the planktonic cells, which was higher than their proportion in biofilms. By contrast, the abundance of *Firmicutes* and *Bacteroidetes* in planktonic cells was lower than that in biofilms.

At the genus level (Fig. 7b), the predominant species mainly included Arcobacter, *Sulfurospirillum*, *Acetobacterium*, and *Citrobacter*. Remarkably, the relative abundance of *Arcobacter* in planktonic cells (91.8 ± 0.3%) for the control group was higher than those for the other groups, followed by *Citrobacter* (4.4 ± 0.3%), *Sulfurospirillum* (2.2 ± 0.4%), and *Acetobacterium* (0.9 ± 0.3%). Moreover, the relative abundance of *Acetobacterium* in biofilms was higher than that in planktonic cells. This phenomenon is consistent with previous reports [14]. The dominant species in biofilms was also *Arcobacter* (65.4 ± 0.2%), followed by *Sulfurospirillum* (16.6 ± 0.6%), *Acetobacterium* (14.7 ± 1.7%), and *Citrobacter* (2.6 ± 0.1%).

## Discussion

Hydrogen is a well-recognized energy carrier that can be easily developed further [34]. Lanchet et al. [35] reported that hydrogen produced on the cathode by water electrolysis is an essential mediator in the microbial electrochemical. Later, Jourdin et al. [36] found that bacterial modification of the electrode surface (possibly via synthesis of Cu nanoparticles) was directly involved in the significant enhancement of the hydrogen production. In MES, H_2_ might be produced in the cathodic chamber by water electrolysis. However, the activation energy of hydrogen evolution reaction without catalyst is relatively high. Therefore, coupling a HER catalyst to a cathode of a MES system to enhance hydrogen evolution might be a promising strategy to improve the MES performance, from the engineering perspective.

The HER electrocatalyst used in this study was Mo_2_C, which is an important member of early transition metal carbides. It has been proven to be a high-performing HER electrocatalyst because it exhibits an electronic structure similar to that of platinum group metals [29, 37]. For the cathode of our MES system, CF was used as the base material and commercially available β-Mo_2_C powder was selected as HER electrocatalyst. Consistent with our designation assumptions, the decoration of Mo_2_C considerably improved hydrogen evolution in our MES systems. The Tafel plot constructed from steady-state polarization measurements is believed to show HER activity from a kinetic point of view. The cathode with 12 mg cm^−2^ Mo_2_C loading achieved the best HER activity, whereas its Tafel slope yielded the lowest value of 0.131 V dec^−1^ (Fig. 2b), followed by the cathodes with 4 mg cm^−2^ (0.215 V dec^−1^) and 2 mg cm^−2^ (0.279 V dec^−1^) Mo_2_C loadings. The bare CF demonstrated the worst electrocatalytic performance in generating hydrogen from the cathode medium, and its Tafel slope reached as high as 0.325 V dec^−1^. The results of H_2_ production rate also further confirmed that the presence of Mo_2_C can improve the hydrogen evolution. The average H_2_ production rate of Mo_2_C–CF (2.29 × 10^4^ mol day^−1^) was 12.7 times than that of CF (1.80 × 10^3^ mol day^−1^) (Fig. 3b).

The SEM images and Bradford assay indicated that the formation of biofilms was improved by Mo_2_C modification of the cathode. A comparison of the SEM images of biofilms on the cathodes with and without Mo_2_C modification showed that the distribution of biofilm on the cathode with 12 mg cm^−2^ Mo_2_C was more extensive (Additional file 1: Fig. S3). Meanwhile, Bradford assay demonstrated the same results. The protein content of the cathode with 12 mg cm^−2^ Mo_2_C was nearly twice as much as that of the control. It is probably due to that the more production of electron carrier, hydrogen, improved autotrophic microbial growth significantly. We evaluated the average H_2_ production rates in MES with Mo_2_C biofilm (12 mg cm^−2^) and CF biofilm on day 14 of MES operation. The average H_2_ production rate of Mo_2_C biofilm reduced to 4.94 × 10^2^ mol day^−1^, while the H_2_ production was almost undetectable in the CF biofilm (Fig. 3b). Compared with the average H_2_ production rates in MES with bare Mo_2_C–CF (2.29 × 10^4^ mol day^−1^) and CF (1.80 × 10^3^ mol day^−1^), almost 98% and 100% of H_2_ produced in MES system with Mo_2_C-modified and unmodified cathode were consumed by autotrophic microorganisms, respectively. Therefore, the biocatalysts in biofilms and planktonic cells acquire electrons more easily from the Mo_2_C-modified MES systems because of the highly efficient electrocatalyzed hydrogen production.

The presence of Mo_2_C also regulates the mixed microbial flora of biofilms and planktonic cells in the MES system. The predominant species in the microbial community of MES systems include *Arcobacter*, *Acetobacterium*, *Sulfurospirillum*, and *Citrobacter.* Compared with the microbial community of the CF biofilm, the content of *Arcobacter* decreased, whereas that of *Acetobacterium* increased in the 12 mg cm^−2^ Mo_2_C biofilm. *Arcobacter* has been reported to be an electrochemically active bacterium that is responsible for electron transfer via an electrode [38, 39], whereas *Acetobacterium* was recognized as the main acetogen in bacterial flora that is responsible for acetate production through the Wood–Ljungdahl pathway [7, 16, 17, 39, 40]. As an electronic carrier, H_2_ played a dominant role in electron transport and considerably promoted electron transfer through a hydrogen-related metabolic pathway because of the decoration of HER catalyst in the MES system. Therefore, electron transfer through *Arcobacter* was relatively reduced, and acetate production depending on H_2_ was strengthened. A similar pattern was observed in planktonic cells. The content of *Arcobacter* in the planktonic cells of the CF group was lower than that in 12 mg cm^−2^ Mo_2_C group. Meanwhile, *Acetobacterium*, which was nearly undetectable in the planktonic cells of the CF group, significantly accumulated in the 12 mg cm^−2^ Mo_2_C group. We also observed that the abundance of *Sulfurospirillum* was increased in 12 mg cm^−2^ Mo_2_C in biofilm (16.7 ± 0.3%) and planktonic cells (6.5 ± 0.1%). *Sulfurospirillum*, which was reported as the microaerophilic bacterium in the cathodic microbial community [39], was responsible for expending the trace amounts of oxygen that diffused from the anode and for maintaining an anaerobic environment in the rest of the community. The abundance of *Sulfurospirillum* in MES with 12 mg cm^−2^ Mo_2_C was higher than that of MES with CF. This phenomenon suggested that more microaerobic bacteria might be needed to maintain anaerobic environment in the MES with 12 mg cm^−2^ Mo_2_C. In addition, *Citrobacter* had been reported as a hydrogen-producing bacterium [39]. We observed that the abundance of *Citrobacter* in the planktonic cells of the 12 mg cm^−2^ Mo_2_C group was considerably lower than that of the CF group because of the electrochemical catalyzed hydrogen evolution. However, this pattern was not as significant as that in biofilm. This phenomenon may be explained by the dominant electron transfer mechanism being MET in planktonic cells and DET in biofilms. Therefore, when HER catalyst was used to enhance hydrogen production, its impact on MET was considerably higher than that on DET. In summary, the decoration of HER catalyst on the cathode remarkably enhanced indirect electron transfer through H_2_ and regulated the composition of microbial flora in a MES system, thereby causing a reduction in the abundance of the electron transfer bacterium *Arcobacter* and the hydrogen-producing bacterium *Citrobacter* and promoting microbial electrosynthesis through the Wood–Ljungdahl pathway, which caused *Acetobacterium* accumulation. The side effect of water electrolysis is the additional requirement of the microaerophilic bacterium, *Sulfurospirillum*, in a MES system with Mo_2_C decoration.

In the present study, coupling an active HER electrocatalyst to a cathode of MES system enhanced the electron transfer rate, thereby promoting an effective CO_2_ reduction process. Consequently, a relatively high maximum acetate concentration of 5.72 g L^−1^ was obtained from the MES with 12 mg cm^−2^ Mo_2_C decoration, which was 2.08 times higher than that of the control. The maximum acetate concentration between our study and those in the literature was incomparable due to the differences in mixed microbial cultures, operation days, and potentials. However, as the reaction goes on, due to the continuous accumulation of acetate, the activity of electroautotrophic bacteria might be inhibited. The volumetric production rate and CE are meaningful for practical industrial use. Therefore, a lot more researches focused on the acceleration of volumetric acetate production rate through material science [41, 42] or separation process of acetate [43], rather than the maximum acetate production. We summarized the key values in Table 1, which indicates that the efficiency that MES systems was obtained via mixed microbial cultures using different cathode materials and potentials. Our MES system with 12 mg cm^−2^ Mo_2_C demonstrated the outstanding production rate of 0.19 g L^−1^ day^−1^, which is higher than those of most reported MES systems but lower than that of the MES system reported by Marshall et al. (1.0 g L^−1^ day^−1^) [17]. For their MES system, the high volumetric production rate might be due to the high packing density of graphite granules (400 g L^−1^) in their MES system, where the packed bed was fully clogged with biocatalysts. However, due to the increased internal resistance in their MES system, the volumetric acetate production rate for certain amount of biocatalysts might not be optimized. Our strategy is to improve hydrogen evolution by electrode modification, the volume rate of acetate production was effectively increased, and the autotrophic microorganism may gain electrons through hydrogen, which effectively reduced internal resistance caused by excessive microorganisms.

**Table 1:** Comparative overview of acetate production in MES with mixed cultures

Cathode material	Ecathode (V vs. SHE)	Current density (A m^−2^)	Volumetric production rate (g L^−1^ day^−1^)	Maximum acetate titer (g L^−1^)	CE (%)	References
12 mg cm^−2^ Mo_2_C	− 0.85	− 5.2	0.19	5.72	64	Current study
CF with fluidized GAC (16 g L^−1^)	− 0.85	− 4.08	0.14	3.9	65	[47]
VITO–CoRE (4 g HCO_3_^−^ L^−1^)	− 0.6	− 0.069	0.14	4.97	45.5	[42]
CF and stainless steel	− 0.78	− 15	0.14	2	22.5	[48]
Graphite stick–graphite felt	− 0.8	− 20	0.14	8.28	–	[43]
CF	− 0.903	− 2.96	0.14	4.7	89.5	[41]
Graphene–nickel foam	− 0.85	− 10.2	0.19	5.46	70	[15]
rGO–CF	− 0.85	− 4.9	0.17	7.1	77	[16]
CF	− 1.26	− 5.0	0.06	1.29	58	[8]
CF and stainless steel	− 0.9	− 10	1.3	0.6	40	[32]
RVC–EPD	− 0.85	− 102	–	11	100	[14]
RVC–nanoweb	− 0.85	− 37	0.03	1.65	70	[23]
Graphite granules	− 0.6	–	1.0	10.5	69	[17]

In general, the development of Mo_2_C–CF cathode is a simple, rapid and effective approach to improve MES efficiency. From the perspective of hydrogen evolution catalysts, we need to develop highly efficient hydrogen evolution catalysts under neutral conditions. However, Mo_2_C, the HER catalyst used in this study, always presents the advantage of hydrogen evolution in acidic conditions in previous reports [29]. A catalyst with a large load is required because the hydrogen evolution effect of Mo_2_C is not optimal under neutral conditions. When we increase the loading of Mo_2_C to 24 mg cm^−2^, we find that Mo_2_C is not strongly adsorbed onto the surface of CF. This condition causes the catalyst to fall off and the detached Mo_2_C was suspended in the MES reaction solution (Additional file 1: Fig. S4), which considerably inhibits the growth of microorganisms, Moreover, the decoration methodology may also be improved, and an in situ growth of HER catalyst may be applied in the future to optimize its catalytic efficiency [29].

## Conclusions

In this study, the Mo_2_C-modified electrode was constructed to demonstrate that decorating HER catalyst on cathode is a promising strategy to improve MES process efficiency. When the loading of Mo_2_C was 12 mg cm^−2^, the volumetric acetate production was twofold of the control (0.19 ± 0.02 g L^−1^ day^−1^) and the acetate concentration reached 5.72 ± 0.6 g L^−1^, within 30 days. The presence of Mo_2_C accelerates the release of hydrogen, regulates the mixed microbial flora and benefits the biofilm growth, and thus improves the CO_2_ reduction rate in the MES system.

## Methods

### Mo_2_C-modified carbon felt (CF) preparation

In accordance with previous reports [44], CF was immersed in 1 mol L^−1^ HCl and 1 mol L^−1^ NaOH for 24 h to remove metal impurities and organics, respectively. Then, CF was washed several times with deionized water to ensure that its surface was clean. Mo_2_C powder (99%; Zhongxin, China) was used as the cathodic catalyst in this study. For catalyst ink preparation, 50 mg, 100 mg, 300 mg and 600 mg of Mo_2_C powder were, respectively, mixed with 5 wt% Nafion solution (DuPont, USA) and ethanol (95%, AR) (*V*_Nafion_:*V*_ethanol_ = 1:10). The mixture was ultrasonically agitated for 30 min to obtain a homogenous cathodic catalyst ink. Then, the as-prepared CF was fully soaked in the ink for 12 h and dried at 80 °C in an oven. This process was repeated four times until the ink was adsorbed completely. From the preceding method, the loading of Mo_2_C on the electrode was 2, 4, 12, and 24 mg cm^−2^. CF without modification was used as control.

### Microbial electrosynthesis experiment

The cathodic chamber was filled with a growth medium, which contained 50 mL PETC salt solution, 10 mL trace element solution, 0.5 g Cys-HCl, 1 g NaHCO_3_, and 10 mL Wolfe’s vitamin solution per liter of deionized water. The PETC salt solution was comprised of the following (per liter of deionized water): 1 g NH_4_Cl, 0.1 g KCl, 0.2 g MgSO_4_·7H_2_O, 0.8 g NaCl, 0.1 g KH_2_PO_4_, 0.02 g CaCl_2_. The trace elements solution was comprised in the following manner: (per liter of deionized water): 2 g Nitrilotriacetic acid, 1.3 g MnCl_2_·4H_2_O, 0.4 g FeSO_4_·7H_2_O, 0.2 g CoCl_2_·2H_2_O, 0.2 g ZnSO_4_·7H_2_O, 0.02 g CuCl_2_·2H_2_O, 0.02 g NiCl_2_·6H_2_O, 0.02 g Na_2_MoO_4_·2H_2_O, 0.02 g Na_2_SeO_3_, 0.025 g Na_2_WO_4_·2H_2_O. The Wolfe’s vitamin solution composed of the following (per liter of deionized water): 2.0 mg Biotin, 2.0 mg Folic acid, 10.0 mg Pyridoxine hydrochloride, 5.0 mg Thiamine·HCl, 5.0 mg Riboflavin, 5.0 mg Nicotinic acid, 5.0 mg Calcium d-(+)-pantothenate, 0.1 mg Vitamin B12, 5.0 mg *p*-aminobenzoic acid, 5.0 mg Thioctic acid. The final pH was adjusted to 7.0. The anodic medium consists of 50 mL L^−1^ PETC salt solution, 6 g L^−1^ NaCl, and 2 g L^−1^ KCl L^−1^ of deionized water. The PETC salt solution, trace element solution, and Wolfe’s vitamin solution were prepared by following previous reports [15].

The H-shaped reactors, with an internal volume of 280 mL in both anode and cathode chambers, were made of glass. A proton exchange membrane (Nafion 117; Dupont Co., USA) was selected to separate the anode and cathode chambers. A titanium mesh with iridium and ruthenium coating (50 mm × 25 mm × 1 mm, length × width × thickness; Baoji Longsheng Nonferrous Metal Co., Ltd., China) was used as a dimensionally stable anode in all the reactors. The Mo_2_C-modified CF (Mo_2_C–CF, 5 cm × 5 cm × 0.5 cm, length × width × thickness) and CF (5 cm × 5 cm × 0.5 cm, length × width × thickness) were used as cathodes in duplicate reactors. A multichannel potentiostat (CHI 1000C, Shanghai Chen Hua Instrument Co. Ltd., China) was used for all the experiments. The cathode was poised with a potentiostat setting of − 0.85 V (vs. SHE). The anodic chamber was continually gassed with N_2_. A magnetic stir plate was installed into the cathodic chamber to ensure homogeneous mixing, and 100% CO_2_ (3 mL min^−1^) was continually gassed into the cathodic chamber. The inoculation mix was enriched as previously reported [16]. Then, 5% (V/V) inoculation was added to each cathodic chamber of all the MES systems used in this study. All the reactors were run for 30 days and maintained at room temperature (25 ± 2 °C).

### Analysis methods

The Mo_2_C structures were investigated via X-ray diffraction (XRD; Rigaku Smartlab 3 kW) analysis with filtered Cu K_α_ radiation (λ = 1.5406 Å) operating at a tube voltage of 40 kV and a current of 40 mA. Diffraction patterns were collected via step scanning within the 2θ range of 10°–80° with intervals of 0.02°. HER measurements were conducted at room temperature in a standard three-electrode electrochemical cell with a potentiostat (Zennium, Germany). All the tests were performed in the aforementioned cathodic medium (pH = 6.8) using Mo_2_C–CF or CF (1 cm × 1 cm × 0.5 cm) as working electrode, while platinum sheet and saturated calomel electrode (SCE) were used as the counter and reference electrode. The system was deoxygenated via continuous purging with ultrapure argon gas (99.999% purity) for 30 min before each measurement. The polarization curves were recorded using linear sweep voltammetry (LSV), shifting from 0.2 to − 1.2 V (vs. SHE), at a scan rate of 0.005 V s^−1^. Subsequently, the polarization curves were replotted as overpotential (*η*) versus the logarithm of current density (log |*j*|) to obtain Tafel plots. The linear portions of the Tafel plots were then fitted to the Tafel equation to obtain the Tafel slope (*b*):

1 $$\eta \, = \, b{ \log }\left| j \right| \, + \, a$$

HER catalyst stability of the Mo_2_C–CF was evaluated by LSV before and after 1000 continuous sweeps of CV between 0.2 and − 1.2 V (vs. SHE), at a scan rate of 0.1 V s^−1^ after stabilization. The H_2_ production rate of bare Mo_2_C–CF, CF, Mo_2_C biofilm (on day 14) and CF biofilm (on day 14) was measured by gas chromatography (GC) apparatus (Hope 9860, China) in the cathode compartment of MES reactors with a potentiostat setting of − 0.85 V (vs. SHE). GC analysis was carried out with thermal conductivity detector and ultrapure carbon dioxide gas (99.999% purity). The Faradaic efficiency was calculated by comparing the amount of hydrogen generated by potentiostatic cathodic electrolysis with calculated hydrogen (assuming 100% FE). On the day 14 of MES operation, the CV of all the reactors was scanned, ranging from 0 to − 1.2 V (vs. SHE), at a scan rate of 0.005 V s^−1^. For CV measurement, the cathode (5 cm × 5 cm × 0.5 cm) was used as the working electrode, whereas the anode and Ag/AgCl were used as the counter and reference electrodes, respectively.

The surface morphologies of the cathode surfaces were studied via scanning electron microscopy with coupled energy-dispersive spectroscopy (SEM–EDS; JSM–5900, Japan). The bacteria attached to the cathode were stabilized following previously described procedures at the end of the experiments [45]. Volatile fatty acid (VFA) was measured using a high-performance liquid chromatography (HPLC) apparatus (Agilent Technologies 1260, USA). Alcohols were detected by a gas chromatography (GC) apparatus (Shimadzu 2010 Plus, Japan). To evaluate the biofilm, protein content was quantified via Bradford assay [46]. The currents were continuously monitored using a precision multimeter and a data acquisition system (Keithley Instruments 2700, USA). Coulombic efficiencies (CE) were calculated as CE = *C*_P_/*C*_T_ × 100%, where *C*_T_ is the total coulombs consumed calculated by integrating the area under the current-versus-time curve (*i*–*t* curve). *C*_P_ is the coulombs found in the product calculated as *C*_P_ = *b* × *n* × *F*, where b is the number of electrons in the product (8 eqmol^−1^), n is the number of moles, and *F* is Faraday’s constant (96,485 C mol^−1^).

### DNA extraction for 16S rDNA illumina sequencing and microbial community structure analysis

Samples from the inoculum, planktonic cells, and biofilm on the Mo_2_C–CF and CF cathodes were collected and preserved at − 80 °C until the extraction of the genomic DNA. Genomic DNA was extracted using PowerSoil^®^ DNA Isolation Kit (MO BIO Laboratories Inc., Carlsbad, CA, USA) following the manufacturer’s protocol. DNA quality was assessed at a ratio of 260/280 nm, using a Nano Drop ND-2000 spectrophotometer (Nano Drop Technologies Inc., Wilmington, USA), and a highly pure genomic DNA (A260/A280 ≈ 1.8) was used only for Illumina high-throughput sequencing by Majorbio (Shanghai, China).

Richness and biodiversity indices were obtained using the mothur software package. Similar sequences were clustered into operational taxonomic units (OTUs) based on 3% dissimilarity. On the basis of these clusters, rarefaction curves, OTUs, Chao1 richness estimations, Shannon diversity indexes, and Good’s coverage were generated in mothur version 1.30.0 for each sample (http://www.mothur.org/wiki/Main_Page) at a cut-off of 0.03 by randomly selecting the minimum sequences of all the samples. BLAST reports of taxonomic classification down to the phylum, class, order, family, and genus levels were performed using mothur based on sequences from the ribosomal database project (RDP) with a bootstrap cutoff of 50%.

## Additional file

**Additional file 1.** Additional information.

## Abbreviations

MES: microbial electrosynthesis
HER: hydrogen evolution reaction
RVC: reticulated vitreous carbon
DET: direct electron transfer
MET: mediated electron transfer
XRD: X-ray diffraction
SEM: scanning electron microscope
SEM–EDS: scanning electron microscopy with energy-dispersive spectroscopy
CF: carbon felt
LSV: linear sweep voltammetry
CV: cyclic voltammetry
CE: coulombic efficiency
VFA: volatile fatty acid
HPLC: high-performance liquid chromatography
OTU: operational taxonomic unit
