# Supplementary material for: Mo2C-induced hydrogen production enhances microbial electrosynthesis of acetate from CO2 reduction
Source: Biotechnol Biofuels. 2019 Apr 1;12:71. doi: 10.1186/s13068-019-1413-z (PMC6442412; doi:10.1186/s13068-019-1413-z)
Supplement: Supplementary file 1 — Additional file 1. Additional information. [file 13068_2019_1413_MOESM1_ESM.docx]

**Additional Information**

**Mo_2_C induced hydrogen production enhances microbial electrosynthesis of acetate from CO_2_ reduction**

Shihao Tian^a,b^, Haoqi Wang^a,b^, Zhiwei Dong^a,b^, Yang Yang^c^, Hao Yuan^c^, Qiong Huang^d^, Tian-shun Song^a,b,c,d^, Jingjing Xie ^a,b,c,e†^

*^a^State Key Laboratory of Materials-Oriented Chemical Engineering, Nanjing Tech University, Nanjing 211816, PR China*

*^b^College of Life Science and Pharmaceutical Engineering, Nanjing Tech University, Nanjing 211816, PR China*

*^c^Jiangsu Branch of China Academy of Science & Technology Development, Nanjing 210008, PR China*

*^d^Jiangsu Collaborative Innovation Center of Atmospheric Environment and Equipment Technology, Jiangsu Key Laboratory of Atmospheric Environment Monitoring and Pollution Control (AEMPC), Nanjing University of Information Science & Technology, Nanjing 210044, PR China*

*^e^Jiangsu National Synergetic Innovation Center for Advanced Materials (SICAM), Nanjing 211816, PR China*

^†^Correspondence and requests for materials should be addressed to J.X. ([xiej@njtech.edu.cn](mailto:xiej@njtech.edu.cn))





Fig.S1 XRD patterns of Mo_2_C.


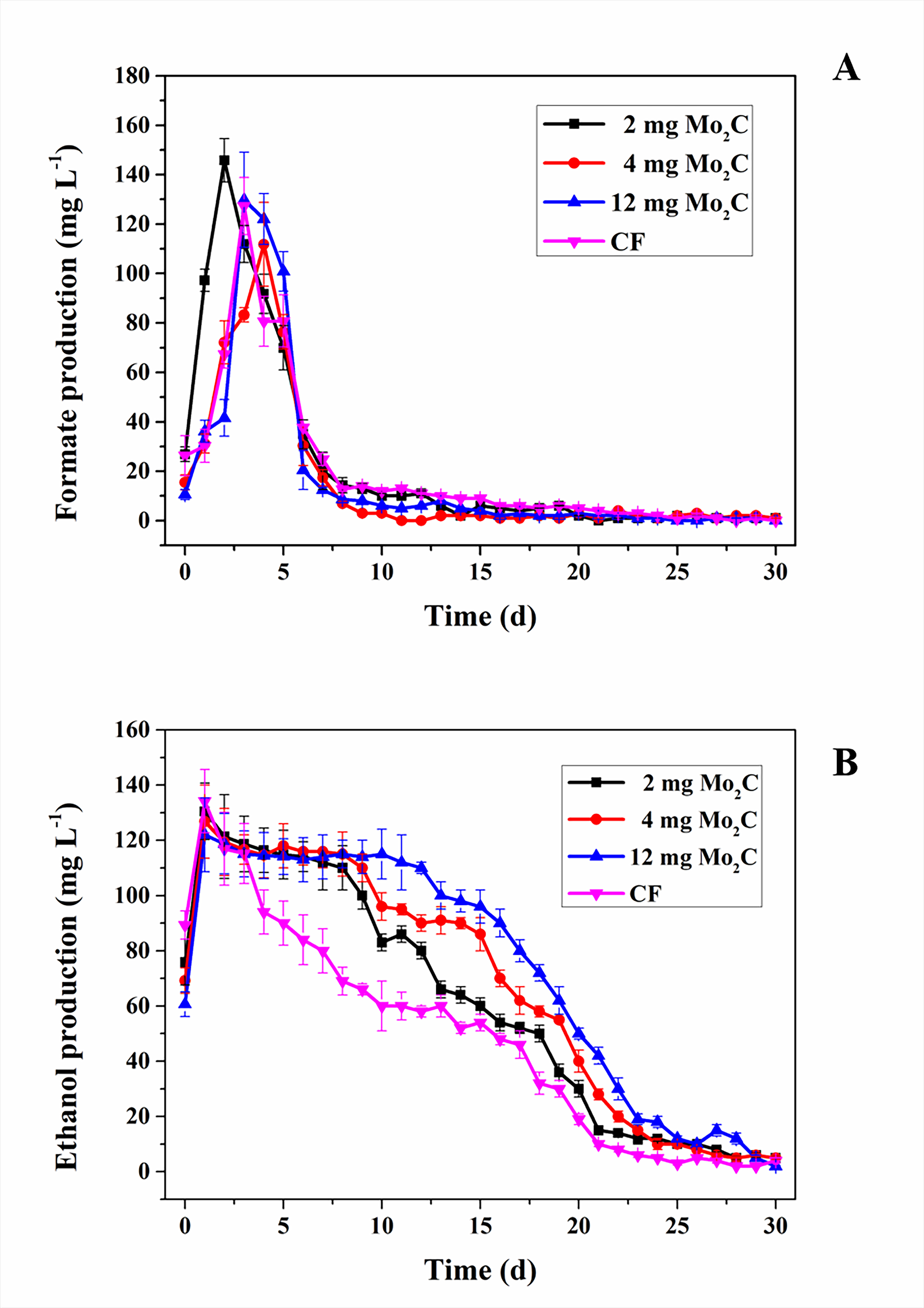


Fig.S2 Formate production (A) and ethanol production (B) of MES with Mo_2_C–CF and CF over 30 days at −1.05 V vs. Ag/AgCl.





Fig.S3 SEM images of biofilm in (A, B) CF, (C, D) 2 mg cm^-2^ Mo_2_C-CF, (E, F) 4 mg cm^-2^ Mo_2_C-CF and (G, H) 12 mg cm^-2^ Mo_2_C-CF at the end of experiment.


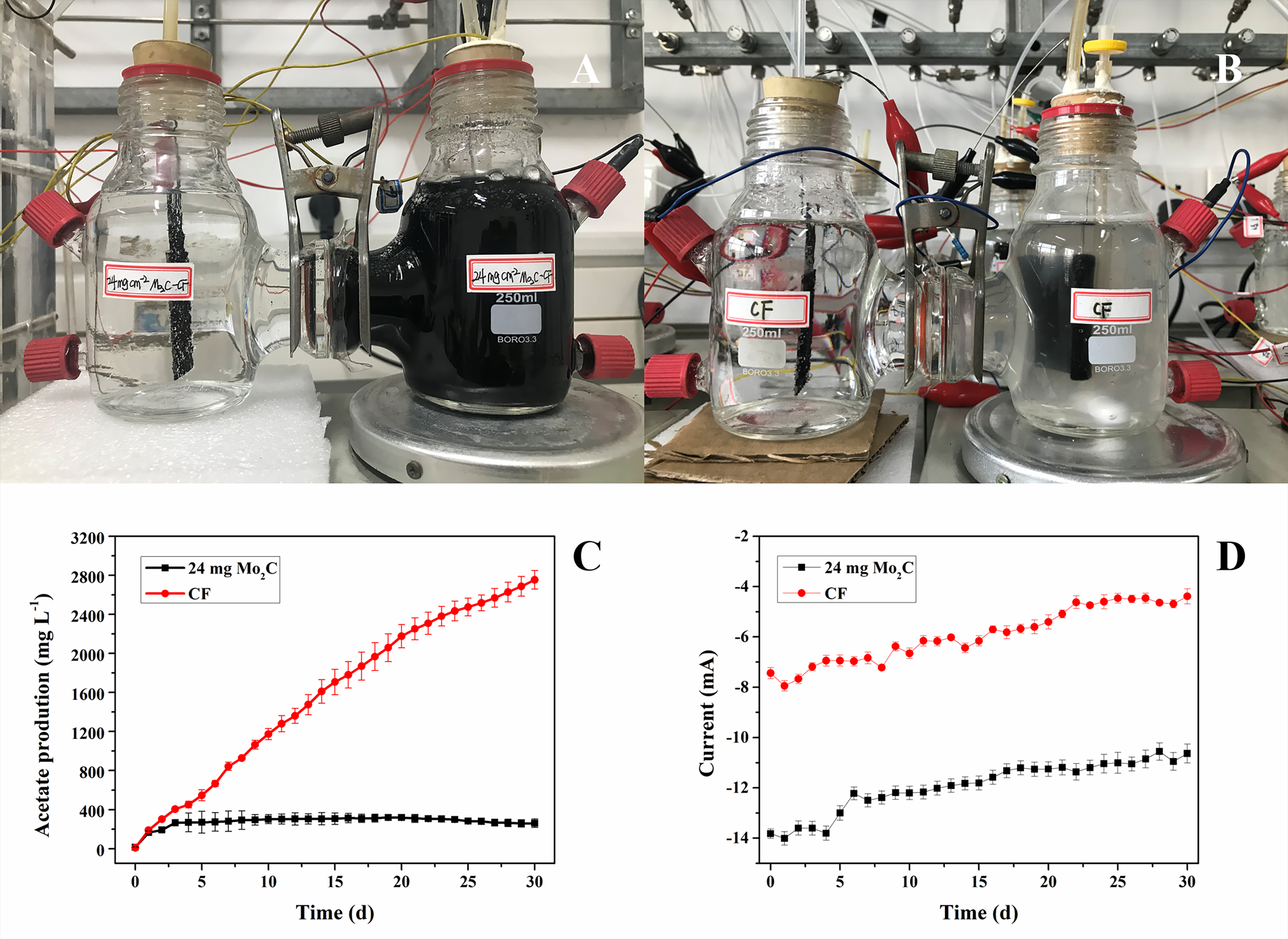


Fig.S4 MES equipment of 24 mg cm^-2^ Mo_2_C–CF (A) and CF (B); acetate production (C) and current (D) of MES with 24 mg cm^-2^ Mo_2_C–CF and CF over 30 days at −1.05 V vs. Ag/AgCl.

Table.S1 EDS spectra of Mo_2_C-carbon felt and Carbon felt

| Element Name | Mo_2_C-carbon felt | | Carbon felt | |
| --- | --- | --- | --- | --- |
|  | At % | Wt % | At % | Wt % |
| Carbon | 88.28 | 75.41 | 87.89 | 84.49 |
| Oxygen | 7.49 | 8.52 | 12.11 | 15.51 |
| Molybdenum | 1.89 | 12.92 | - | - |
| Fluorine | 2.34 | 3.16 | - | - |
